# News from South Africa

**Published:** 1920-06-12

**Authors:** 


### NEWS FROM SOUTH AFRICA.

### The Hospital Shortage.

THE great shortage of hospital accommodation at Capetown is occasioning much comment. Last year the "admission refusals" numbered 1,150—651 European and 499 coloured patients. Overcrowding in the city being at its height, the hospital shortage is being very acutely felt.

### Denis Buxton Memorial Hospital at Capetown.

IN the presence of the Governor-General (Lord Buxton), and the Prime Minister (General Smuts), the opening of the Denis Buxton Memorial Hospital for Children took place at Woodstock, a suburb of Capetown, in the middle of April. The building has been erected by Lord and Lady Buxton, and relatives and friends in South Africa and England, in memory of Mr. Denis Bertram Sydney Buxton, 2nd Battalion Coldstream Guards, who left South Africa to enlist for active service and was killed in action in Flanders on October 19, 1917. The hospital will provide for fourteen European children and will be capable of extension. It is the first hospital for children in the Union of South Africa. The building was declared open by General Smuts, who paid a tribute to the memory of the late Lieutenant Buxton, "the exact manner of whose death no one would ever know."

### Death of Dr. S. R. Savage.

Dr. R. S. Savage, senior visiting physician to the Pretoria Hospital, and a member of the Victoria Cottage Hospital Committee, died at Pretoria on April 27.

Dr. Savage had the reputation of being a skilful obstetrician. He was born and educated at Rondebosch, near Cape Town, and qualified as a medical practitioner at Edinburgh. Under Lord Milner's administration Dr. Savage became railway medical officer at Pretoria, and held the appointment, together with his own private practice, until his death. He was at one time president of the Pretoria branch of the British Medical Association. In 1908 Dr. Savage was mayor of the capital. Many medical men and nurses attended the funeral. Dr. Savage leaves a widow and family.

### The Somerset Hospital, Capetown.

IN memory of the late Major Brydon, D.S.O., of the South African Heavy Artillery, a bed has been dedicated in the Milner Ward of Somerset Hospital, Capetown, on Tuesday, April 13, and a commemorative tablet was unveiled by Mr. Advocate Close, K.C., M.L.A. The tablet bore an inscription, which ended with the words :

"Glad did I live, and gladly die,
And I laid me down with a will."

### Personalia.

COLONEL SIR EDWARD THORNTON, formerly in charge of the South African Military Hospital at Richmond, assumed duty on April 1 at Orange Buildings, Bloemfontein, as chief assistant to the Medical Officer of Health for the Union, Dr. Mitchell.

The choice of a successor to Colonel Stock, C.B., late Director of Medical Services, lies, it is stated, between Colonel C. H. Muller, D.S.O., of Montagu, Cape Province, who served in German South-West Africa, and was afterwards in command of the South African Field Ambulance in East Africa, and Major F. H. Welch, O.C. Roberts' Heights Hospital, Pretoria. Major Welsh served overseas.

